# Can “Functional Sweetener” Context Increase Liking for Cookies Formulated with Alternative Sweeteners?

**DOI:** 10.3390/foods10020361

**Published:** 2021-02-07

**Authors:** Soo-Hyun Lee, Seo-Youn Choe, Ga-Gyeong Seo, Jae-Hee Hong

**Affiliations:** 1 Research Institute of Human Ecology, Seoul University, Seoul 03080, Korea; jinlee0123@gmail.com; 2 Department of Food and Nutrition, College of Science and Technology, Kookmin University, Seoul 02707, Korea; yooon35@gmail.com; 3 Department of Food and Nutrition, College of Human Ecology, Seoul University, Seoul 03080, Korea; tjrkrud94@snu.ac.kr

**Keywords:** alternative sweetener, context, liking, cookie, health-promoting effect

## Abstract

Various strategies for replacing sugar with naturally derived sweeteners are being developed and tested. In this study, the effect of the “functional sweetener” context, which is created by providing health-promoting information, on liking for the sweeteners was investigated using a cookie model system. Cookie samples were prepared by replacing the sugar of 100% sucrose cookies (control) with phyllodulcin, rebaudioside A, xylobiose and sucralose either entirely or partly. The sensory profile of the samples was obtained using descriptive evaluations. Hedonic responses to cookie samples were collected from 96 consumers under blind and informed conditions. Replacement of 100% sucrose with rebaudioside A or phyllodulcin significantly increased bitterness but replacement of 50% sugar elicited sensory characteristics similar to those of the control. Although the “functional sweetener” context did not influence overall liking, liking for the samples was more clearly distinguished when information was provided. Consumers were segmented into three clusters according to their shift in liking in the informed condition: when information was presented, some consumers decreased their liking for sucralose cookies, while other consumers increased or decreased their liking for sucrose cookies. Results suggest that the influence of information varies among individual consumers and that cognitive stimulation, such as health-promoting information, affects liking.

## 1. Introduction

Excessive consumption of sugar can cause obesity, diabetes, cardiovascular disease and dental caries [1]. To reduce sugar consumption, the amount of sugar in consumed products can be reduced or replaced with a low-calorie alternative sweetener [2]. The demand for such sweeteners, particularly those that endow consumed products with sensory characteristics similar to those provided by sugar, is therefore constantly increasing. Low-calorie alternative sweeteners can be divided into two categories according to their sweetness potency: intense sweeteners and bulk sweeteners [3]. Intense sweeteners, which have strong sweetening power, reduce calorie intake because they produce sweetness similar to that of sugar even in small amounts [4]. Sucralose is an artificial intense sweetener produced by chlorination of sucrose [5], 600 times sweeter than sucrose with a sugar like sweetness profile [6]. However, due to health awareness about artificial sweetener, efforts have been made to develop and commercialize natural intense sweeteners [7]. One such natural intense sweetener is rebaudioside A, which is a noncaloric substance extracted from Stevia rebaudiana Bertoni [8]. Rebaudioside A is 250–450 times sweeter than sugar and imparts less off-flavor compared to other steviosides but it elicits bitterness and astringency at high concentrations [9]. Phyllodulcin, an isocoumarin derivative extracted from hydrangea (Hydrangea macrophylla var. thunbergii), has been recently studied as a potential intense natural sweetener because of its high relative sweetness (400–800) and health-promoting effects, which include antifungal, antiallergic and antidiabetic effects [10,11].

Bulk sweeteners, which include sugar alcohols and oligosaccharides, have a lower sweetness than sugar but are low in calories as they are not digested and absorbed by the body [12]. Xylitol, a sugar alcohol produced from xylan [13], has a relative sweetness of 0.7–0.8. Xylobiose, a dimer of xylose, is another functional bulk sweetener; its sweetness is 30% that of sugar. Besides being low in calories, xylitol and xylobiose has several health-promoting effects such as cariostatic effects, hyperglycemic control, prebiotic effects [14].

The sweetness profile and physicochemical stability during processing are key factors for the successful application of an alternative sweetener in a food [15]. The biggest challenges in such applications, especially when replacing sugar with alternative sweeteners, are changes in taste and texture since they are determinants of the consumer’s hedonic response [16]. Alternative sweeteners have sweetness characteristics that differ from those of sucrose, for example, onset and persistence of sweetness, unpleasant flavors such as bitterness and metallic sensation and flavors other than sweetness [17]. Importantly, before tasting the food, consumers have an expectation, based on previous experience, of what the food will taste like. Whether the actual perception matches the expectation influences the consumers’ perceptions and decisions [18,19]. For example, if the expectation is not met by the actual experience, the consumer may reject the product [20]. Siró et al. [21] reported that, if a negative change occurs in the sensory properties of food when a functional ingredient is added, it is likely to induce aversion in the consumers. In fact, many consumers believe that it is impossible to produce healthier products without compromising sensory properties [22]. Markey et al. [23] recently reported that a high percentage of consumers preferred conventional products to those with reduced sugar in a sweet food product category.

Consumers’ reactions to food are influenced not only by their sensory properties but also by food consumption contexts [24]. Context refers to a “set of events and experiences that are not part of the reference event but have some relationship to it” [25]. Contextual effects originate either from variables comprising the stimulus itself (“intrinsic”) or from variables external to the target stimulus (“extrinsic”). One extrinsic variable that creates a contextual effect is information [25]. For instance, consumer interest in the health benefits of foods have encouraged researchers to focus on the effects of “healthy food” or “functional food” contexts that are informed by health-related information [26,27,28,29,30]. Hedonic response or behavioral disposition can be altered by various combinations of sensory experience and health-related claims [31]. Therefore, even if the sensory properties of food change due to the use of alternative sweeteners, if information on benefits (such as the health-promoting effects of alternative sweeteners) is provided, consumer acceptance of negative properties may increase or positive effects on liking or choices may be observed.

In previous studies, the effects of health-related information on liking have not been consistent. Some studies [26,27,28] have reported that consumer preference for products decreases when low-sodium and low-fat information is provided. For example, when low-salt crackers were presented in a “reduced salt” context, consumers’ expected and actual likings decreased significantly [27]. Norton et al. [28] found that placing a low-fat label on chocolate significantly decreased consumers’ expected liking. In contrast, other studies have reported the positive effects of providing health information. Information on sugar reduction and the use of natural sweeteners, for example, has significantly increased the overall acceptability of orange and pomegranate juice [32]. When information on health-promoting effects is provided, negative characteristics such as bitter, astringent and herbal flavors were interpreted as the presence of functional compounds, which reduced rejection and increased acceptance of the foods [29]. These inconsistent results show that the effects of health-related information on liking can vary depending on the type of product tested [30], as well as consumer, attitude, belief [33] and individual interest in health [26]. Such information is processed through wide ranging cognitive integration with personal factors such as age, gender, attitude and belief, as well as social norms and other extrinsic contextual factors that influence affective responses and behaviors [34]. Individuals react only to information that they deem important to them and tend to ignore the rest [35]. Therefore, it is assumed that the interaction between sensory characteristics and information varies across individuals depending on their personal traits. Age and gender are the most frequently studied individual traits that can affect the processing of health information [36]. In addition, dietary habits, degree of health orientation or interest in health [37,38] and the level of knowledge about health information [39] have been studied as potential moderating variables for the effect of health-related information on liking. It is assumed that individual responses to sugar substitutes are important aspects of the overall effect of health-related information on liking for foods containing sugar substitutes.

In the present study, cookies were selected as a model system to investigate consumers’ hedonic responses to a natural alternative sweetener. Cookies were chosen because (1) they are one of the sweet foods in which sugar is a major ingredient that determines the sensory profile, (2) sugar contributes to sensory qualities of cookies other than sweetness (e.g., sugar tenderizes cookie texture by inhibiting gluten development and endows a light texture by entrapping air during the creaming process) and (3) sugar develops the brown color and characteristic flavors of cookies through thermal reactions such as the Maillard reaction and caramelization [40]. Therefore, cookies were considered as an appropriate model system for comprehensively evaluating the effects of sugar substitution on food.

Specifically, the present study was conducted to understand how providing information on the health-promoting effects of natural alternative sweeteners moderates the influence of sugar replacement on liking, with cookies used as a model system. To achieve this goal, this study attempted to identify the sensory profiles of cookies in which sucrose was replaced with different alternative sweeteners and to determine the characteristics that influence the liking. Furthermore, the question of whether the “functional sweetener” context, formed by providing information on the health-promoting effect of sweeteners, could offset the influence of sensory characteristics and improve acceptability was investigated. Indeed, the results showed that developing a “functional sweetener” context by providing information on the health-promoting effects of alternative sweeteners improved liking to some extent by giving consumers a positive impression.

## 2. Materials and Methods

### 2.1. Samples

#### 2.1.1. Sweeteners

Eight cookie samples were prepared by replacing sucrose with five alternative sweeteners and their combinations (Table 1). Phyllodulcin, rebaudioside A and xylobiose were used as natural alternative sweeteners derived from plants, while sucralose (known to have the sweetness profile most similar to sugar among artificial sweeteners) was included for comparison. The amount of each sweetener was determined by establishing the sweetness equal to that of 15.33% sugar cookies using the 2-alternative forced choice method (2-AFC). Since xylobiose is known to have a daily intake allowance of 0.7–7.5 g, it was added to cookies as a mixture with sugar (xylobiose:sugar = 7:93) to meet the daily intake allowance. Based on preliminary experiments, as the concentration of phyllodulcin and rebaudioside A increased, a stronger bitter aftertaste was detected and sweetness was suppressed. Therefore, it was not possible to determine the concentration that generated sweetness equal to 15.33% sucrose cookies through 2-AFC. Instead, the concentration of phyllodulcin and rebaudioside A that generated the same sweetness as that of 7.66% sucrose cookies, that is, half the concentration of 15.33%, was determined and this concentration was doubled in the final cookies. In addition, a mixture of phyllodulcin and xylitol in a 1:1 ratio was prepared to compare the sensory characteristics of cookies in which a phyllodulcin and xylitol mixture was used to cookies in which 50% and 100% of the sucrose was replaced with phyllodulcin.

#### 2.1.2. Sample Preparation

**Figure 1:**
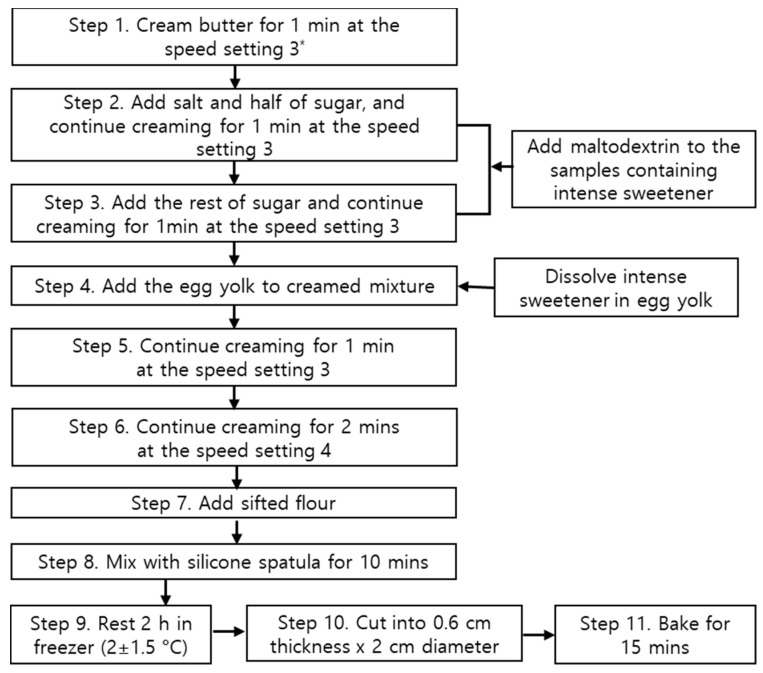
Cookie preparation procedure. * Food Mixer (HB-125, Sanshui Hop Shing Metal & Plastic Manufactory Ltd., Foshan, China).

The ingredients and procedure for cookie preparation are shown in (Table 2 and Figure 1), respectively. A decrease in volume of cookies due to the replacement of sugar with intense sweeteners, such as sucralose, phyllodulcin and rebaudioside A, was compensated for by adding maltodextrin (ES Food, Gyeonggi-do, Korea), which is a bulking agent commonly used in zero- or low-sugar bakery products during the creaming process (steps 2 and 3; Figure 1). Intensive sweeteners were dissolved in egg yolk for 3 min (step 4; Figure 1) to ensure complete incorporation. When replacing sugar with xylobiose or xylitol, maltodextrin was not used and xylobiose or xylitol were added in the creaming process (steps 2 and 3) in the same manner as sugar. After completing the mixing process, the cookie dough was divided into quarters, wrapped in a polypropylene film and allowed to rest for 2 h at 2 ± 1.5 °C. The rested cookie dough was rolled and cut into a circular shape (diameter: 2 cm; thickness: 0.6 cm) and then baked for 15 min at 170 °C. The cookie samples were stored in a sealed plastic package at −16 ± 2 °C until their use in the tests.

The cookie samples were equilibrated at the room temperature (22 ± 2 °C) for 2 h before the evaluation. For appearance evaluations, samples were presented on a disposable white paper plate (10 cm in diameter). For tasting, the samples were presented in a black disposable polystyrene container (70 mm in diameter × 40 mm depth) to minimize color bias. The samples were coded with a three-digit random number. Cucumber sticks (3 cm × 1 cm × 1 cm) and filtered water at room temperature (22 ± 2 °C) were provided for palate cleansing and mouth rinsing, respectively.

### 2.2. Sensory Profiling

#### 2.2.1. Panel Recruiting and Training

Descriptive analysis was conducted by ten panelists (three men and seven women, aged 20–29). The panelists were recruited by posting recruitment flyers on the campus of Kookmin University (Seoul, Korea). Panelists were selected based on the results of the screening tests proposed by Lawless and Heymann [41]: the basic taste test, aroma description test, sweet taste ranking test and sample descriptive test. At the first training session, panelists were briefly introduced to the purpose of the test and principles and general practices of descriptive analysis. In the following sessions, the panelists developed the descriptors and their definition, reference materials (Table 3) and standardized tasting and rinsing protocols by consensus. Four practice tests were then conducted to check panel performance. If a panelist’s data was not consistent with the entire panel or not reproducible, additional training was provided. The training sessions were conducted three or four times a week for 2 months and lasted 1 h per session. The research protocol was approved by the Institution Review Board at Kookmin University (IRB no. KMU-201509-HR-075-P1-C1). Participants reviewed and signed the IRB-approved informed consent form prior to training.

#### 2.2.2. Test Procedure

The descriptive analysis was performed using a generic descriptive analysis procedure [41]. The test was conducted in a sensory tasting room at Kookmin University. Eight samples were presented in a monadic manner following Williams Latin square design [42]. After evaluating four samples, the next four samples were evaluated after a 5 min break. Panelists were instructed to cleanse their palate by chewing cucumber sticks and then rinse their mouth with filtered water between each sample evaluation. To minimize color bias, the odor, taste, flavor, aftertaste and texture attributes were evaluated under red light. Appearance was evaluated in a natural light setting (North sky day light, CIE-F7, 6500K, Multi Light Booth, Super Light VI; Bowoo Engineering Co., Ltd., Gunpo, Korea) separately from the tasting test. The intensities of sample attributes were rated on a 16-point category scale (0: imperceptible, 1: very weak, 15: very strong). Panelists were allowed to retaste samples and modify previous scores if necessary. The test was repeated four times. Panelists were required not to eat, drink or take oral care from 1 h before the test.

### 2.3. Consumer Test

The participants were recruited from the consumer panel pool of Kookmin University’s Sensory Science Lab or from the local community on campus or near the university. In total, 96 participants (aged 19–50 years old) who did not have problems eating sugar, alternative sweeteners, butter, eggs and cucumbers were recruited. Food intake (except water) was prohibited 30 min before the test and the use of perfumes and cosmetics was restricted.

**Figure 2:**
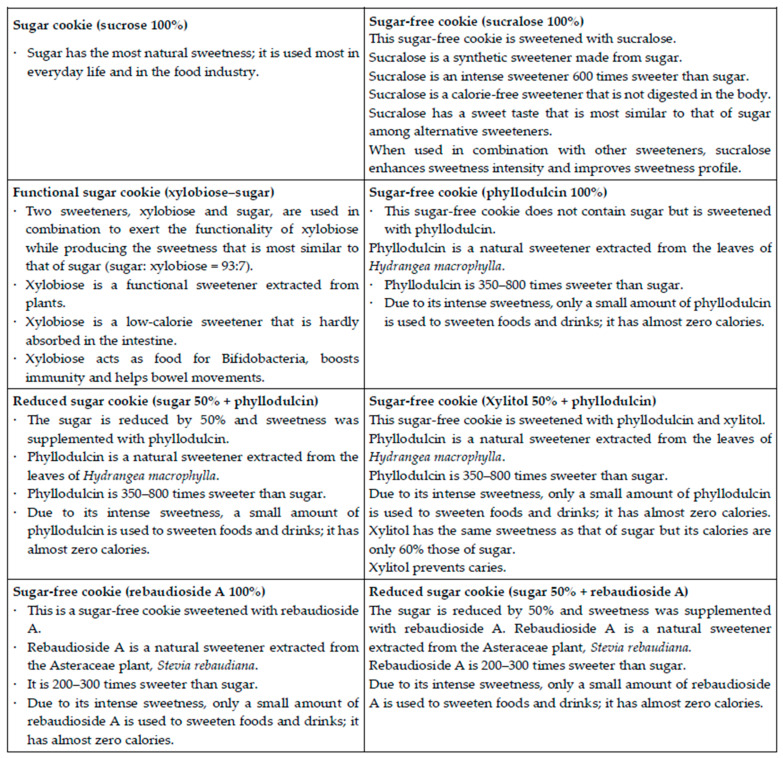
Information cards presented in the informed condition test.

The samples were prepared and presented in the same manner as for the descriptive analysis. The test was conducted in two sessions. In the first session, the test was conducted under blind conditions. The second session was conducted one week after the first session and the participants were provided with information about each sample (i.e., the informed condition). Information was provided as a written message on a card (Figure 2); it consisted of the name of the sweetener and its major health-promoting effects. Before the test, the content of the message had been verified by experts in food science and nutritional science. In addition, the tone of message was confirmed to be neutral and similar across samples in a preliminary test (in a small group interview setting).

In each session, participants evaluated overall liking, appearance liking, flavor liking, texture liking and purchase intent. Likings were rated on a 9-point category scale (e.g., 1 = dislike very much; 9 = like very much) and the purchase intent was rated on a 7-point Likert scale (e.g., 1 = strongly disagree; 7 = strongly agree) [43]. The test was conducted in a monadic manner following Williams Latin square design. Cucumber sticks and filtered water (22 ± 2 °C) were provided for palate cleansing and mouth rinsing, respectively. After completing the test, the panelists answered questions about their demographic information and cookie-eating habits.

### 2.4. Statistical Analysis

#### 2.4.1. Descriptive Analysis

ANOVA was conducted to test for significant differences in the sensory attributes among samples. Panel, sample, replication and their two-way interactions were included in the ANOVA model. Tukey’s Honestly Significant Difference (HSD) test was conducted as a post-hoc analysis. A principal component analysis (PCA) was performed to obtain sensory representations of samples and sensory attributes. The global sensory differences between samples were visualized with confident ellipses using the bootstrap technique (500 iterations) and evaluated by *P* values related to Hotelling’s *T*^2^ test.

#### 2.4.2. Consumer Test

A two-way ANOVA was conducted to assess the significance of the effects of sample and information on liking ratings and purchase intent. The model included sample and information as main factors as well as their secondary interactions. Tukey’s HSD tests and paired t-tests were conducted as post-hoc analyses. Overall liking, as a supplementary variable, was projected onto the PCA map constructed from the descriptive analysis data to explore drivers of liking under different information conditions. In addition to ANOVA, the influence of information on liking was assessed by using cluster analysis to segment participants according to the pattern of changes in liking after presentation of information. Cluster analysis was conducted on the values obtained by subtracting overall liking scores in the blind condition from those in the informed condition. Paired t-tests, ANOVA and Tukey’s HSD tests were used to determine the significance of differences in acceptance changes among samples for each cluster. Additionally, a chi-square test was performed to identify significant relationships between demographic variables and cluster formation. The level of significance for all analyses was *p* < 0.05. ANOVA, Tukey’s HSD tests, cluster analysis and paired *t*-tests were performed using SPSS version 18.0 (SPSS Inc., Chicago, lL, USA). PCA and Hotelling’s T^2^ test were conducted using SensoMineR and FactoMineR packages in R version 4.0.2 [44].

## 3. Results and Discussion

### 3.1. Descriptive Profiling

**Figure 3:**
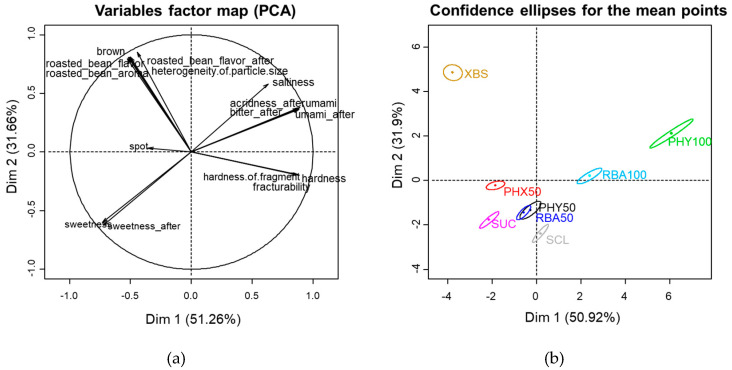
Principal component loading of the sensory attributes (**a**) and sample scores (**b**) from eight sweetener cookies on Dim 1 and Dim 2.

From the descriptive analysis, 16 characteristics were derived (Table 4). According to the multivariate correlation between samples and properties derived by PCA, 82.98% of the total variability was explained by Dim 1 (51.26%) and Dim 2 (31.66%) (Figure 3a). In the positive direction of Dim 1, umami, umami aftertaste, acridness aftertaste, bitter aftertaste, hardness, fracturability and hardness of flakes were highly loaded, whereas sweetness and sweetness aftertaste were highly loaded in the negative direction. Phyllodulcin (PHY100) and rebaudioside A (RBA100) cookies were clearly distinguished from the other samples on Dim1 (Figure 3b).

The positive direction of Dim 2 was loaded with brown color, aroma, flavor and aftertaste of roasted bean powder and heterogeneity of particle size. Sucrose (SUC, control), Phyllodulcin + Xylitol (PHY50) and Rebaudioside A + Sucrose (RBA50) samples were located close to the control (SUC) and showed a similar sensory profile.

Samples in which sugar was replaced with PHY100 and RBA100 were significantly less sweet than sugar cookies (Table 4). For PHY100 and RBA100, there was no sweetening effect due to sugar; the remarkable bitterness suppressed the sweetness [45], so the sweetness was judged weak. On the other hand, when 50% of the sucrose was replaced by phyllodulcin or rebaudioside A, sweetness was either not significantly changed or decreased only slightly. In addition, residual bitterness, umami and acridness of PHY50 were significantly weaker than those of PHY100 but not significantly different from those of SUC. Figure 3b also shows that PHY50 was located close to SUC, indicating that their sensory characteristics were globally similar. Results show that use of phyllodulcin or rebaudioside A in combination with sucrose masked bitterness and acridness, indicating that a substantial amount of sucrose can be replaced without impairing sensory properties.

Xylobiose + Sucrose (XBS) cookies were distinguished from the other samples by their distinctive sensory profile, including its aroma and flavor of roasted bean powder, heterogeneous particle size and strong brown color. The development of a dark exterior color was likely due the Maillard reaction, since xylobiose is a reducing disaccharide [46].

The samples replaced with 50% or 100% intense sweeteners were significantly harder, more fracturable and had more heterogeneous particles than SUC cookies. This is likely due to the addition of maltodextrin as a bulking agent. Maltodextrin binds water to form gels and thereby reduces free water, which is required for hydration of gluten and increases hardness [47]. The textural characteristics of Phyllodulcin + Xylitol (PHX50) and XBS samples were not significantly different from those of SUC samples. The hygroscopic nature of xylitol [48] when used with phyllodulcin might have counteracted the hardening effect of maltodextrin by increasing moisture content. The similarity of the textural attributes between XBS and SUC cookies may be attributable to the fact that only 7% of the sucrose was replaced by xylobiose in the XBS variety; thus, the remaining 93% sucrose likely determines the texture of these cookies.

### 3.2. Consumer Acceptance

#### 3.2.1. Consumer Characteristics

The consumer panel comprised 38.5% men and 61.5% women (Table 5). Approximately half (52.1%) of the panel was in their 20 s, while those in their 30 s and 40 s accounted for 27.1% and 20.8%, respectively. Approximately half of the panel was students, while the other panelists included homemakers, office workers and part-time workers. Most of the panelists (77.1%) considered flavor important when purchasing food. The majority (77%) also consumed cookies more than once a month. Only 25% of the panel had not tried weight control, whereas the other panelists either had tried weight control before (58.3%) or were currently undertaking weight control (16.7%). Low-sugar foods were not novel to 62.5% of the panelists and most panelists (93.8%) were in a healthy condition.

#### 3.2.2. Consumer Liking and Purchase Intent

There were significant (*p* < 0.05) differences in liking and purchase intent among the samples (Table 6). When both information conditions were considered, SUC, RBA50, PHY50 and SCL cookies were significantly preferred over PHY100 and RBA100 alternatives in terms of overall liking, flavor liking and texture liking (Table 7). The sample with the highest purchase intent was SUC, followed by RBA50, PHY50 and XBS.

The “functional sweetener” context, formed by providing health-promoting information, had a significant effect on purchase intent (Table 6). Provision of information increased purchase intent for all samples (Table 7). Provision of information showed a marginal but nonsignificant effect (*p* = 0.08) on overall liking. None of the interactions between the samples and the information was significant. We presume that the influence of the information was moderated by the magnitude of the sensory difference between the samples. Carrillo et al. [49] found that panelists discriminated samples mainly based on the sensory characteristics and reduced-calorie information only partially contributed to discrimination when the sensory differences among the samples were large. In addition, Felipe et al. [32] reported that information on nutritional characteristics and reformulation strategies of products could have a positive effect on consumer sensory perception when sensory differences between the products are not obvious. Furthermore, when the sensory differences between samples are small, liking for the samples is mainly influenced by the information provided [50]. Since substitution with alternative sweeteners in the present study caused significant and apparent differences in sensory characteristics among the samples, it is assumed that the sensory properties of the samples had a greater influence on liking than did the provision of information.

Although the effect of “functional sweetener” context was not significant when all samples were considered, it is difficult to conclude that providing information on health-promoting effects did not affect liking when closely examining the results obtained in the blind and informed conditions. Overall, the liking in the informed condition was higher than that in the blind condition. In particular, the hedonic ratings for SUC, PHX50, RBA50 and RBA100 samples significantly increased after information was provided (Table 7). In addition, information about sugar increased overall liking, liking for appearance and liking for texture for SUC cookies. PHY100 cookies were least liked among the samples; nevertheless, liking for their flavor and texture as well as purchase intent increased significantly with provision of information. This suggests that the “functional sweetener” context prompts consumers to more readily accept undesirable sensory qualities caused by sugar replacement.

Information about the health-promoting effects of food is thought to increase consumer acceptance via the halo effect, that is, by creating an overall positive impression [51]. However, liking for Sucralose (SCL) cookies decreased when information was provided. This seems to be because the sucralose used in these cookies was a synthetic sweetener, which may have given a negative impression to consumers in comparison to other samples, which contained plant-derived sweeteners considered “natural.”

The impact of providing information is also indirectly demonstrated by the panelists’ higher discrimination when information was provided. Park and Hong [50] reported that the overall liking for soymilk sweetened with an alternative sweetener increased and samples were better discriminated (in terms of acceptance scores) when information was provided. Providing information related to “low energy” or “natural sweetener” may motivate consumers to give greater importance to the sweetness, bitterness and aftertaste that are emphasized in products containing alternative sweeteners [52].

#### 3.2.3. Drivers of Liking

**Figure 4:**
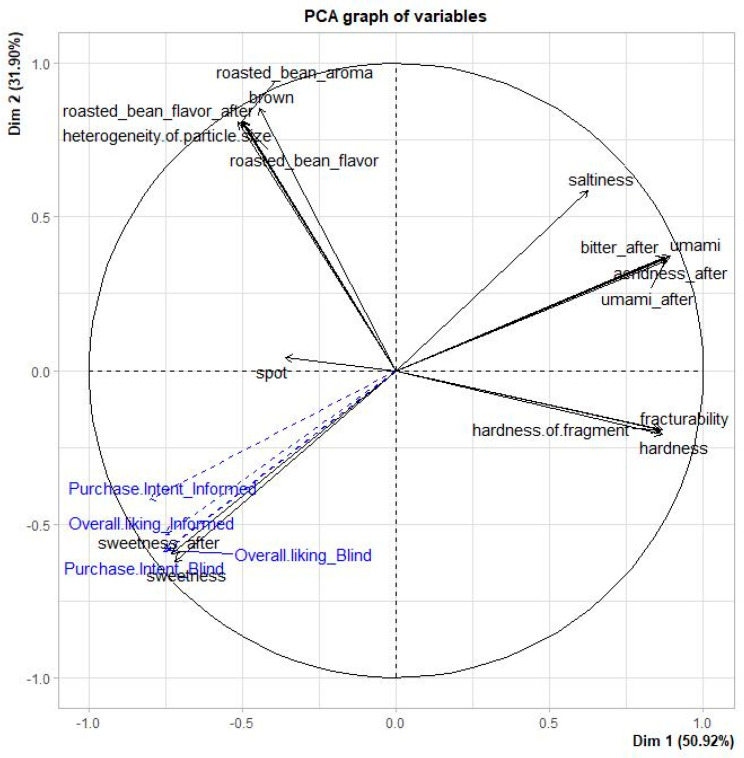
Drivers of liking depending on the information condition.

To identify the drivers of liking under different information conditions, overall liking and purchase intent were projected onto the sensory space constructed with descriptive analysis data as supplementary variables (Figure 4). Irrespective of information conditions, overall liking and purchase intent were positively correlated with sweetness and sweetness aftertaste. On the other hand, umami and aftertaste of umami, bitterness and acridness, which were located on the opposite side of the space, negatively contributed to liking and purchase intent. This result confirms that the sweetness or aftertaste of sweetness determines the hedonic response and purchase intent for low-sugar products [16]. Cookies are a food considered inherently unhealthy [53]; therefore, the health-promoting effects of alternative sweeteners seemed to be of low importance in determining the liking and purchase intent of cookies. Indeed, Torres-Moreno et al. [18] reported that provision of health and functionality information did not significantly affect acceptance of desserts, which are mainly aimed at providing pleasure rather than being healthy.

Additionally, it is presumed that the influence of the “functional sweetener” context was limited because individual consumers’ characteristics affected its interpretation. The effect of health-related information on liking may depend on the consumer’s attitudes toward or beliefs about the food item [54] and/or the individual’s interest in their health [26]. Thus, even in the present study, the “functional sweetener” context might have operated in different directions depending on the individual. Since responses exhibited a multimodal distribution, a clear trend might not have been shown when all responses were aggregated and analyzed. Therefore, an additional attempt was made to determine whether the test participants were subdivided into clusters according to their response to the context of “functional sweetener”.

### 3.3. Consumer Segmentation According to Acceptance Shift under the Informed Condition

According to the tendency to shift their liking for cookie samples in the blind condition when subjected to the informed condition, participants were segmented into three clusters (Table 8). Cluster 1 showed a particularly negative reaction to information about sucralose and phyllodulcin in the presence of information. Overall liking of SCL, PHY100 and PHY50 cookies significantly decreased when the “functional sweetener” context was formed, whereas liking for PHX50 cookies significantly increased. This seems to be because participants positively responded to the health-related information on xylitol. Cluster 2 responded more positively to sugar-containing samples even when less information on health-promoting effects was provided for SUC cookies. In Cluster 2, the liking for SUC, XBS, PHY50 and RBA50 samples significantly increased, whereas liking for PHY100 and RBA100 samples was unaffected by the information. Cluster 3 positively reacted to the information on phyllodulcin; however, liking for the cookies decreased in the informed condition when phyllodulcin was mixed with other sweeteners.

Chi-square analysis showed that there was no significant difference in demographic factors and cookie-eating habits among clusters (for age, χ^2^_4, 0.05_ = 8.866, *p* = 0.065; for gender, χ^2^_2, 0.05_ = 1.840, *p* = 0.399; for low-sugar food purchase experience, χ^2^_2, 0.05_ = 0.397, *p* = 0.820; for purchase intention of low-sugar food, χ^2^_4, 0.05_ = 2.324, *p* = 0.676). It was initially hypothesized that demographic factors such as age, gender, low-sugar food related attitudes could moderate the effect of information based on previous findings [26,36,37,38]. However, the results of the present study did not support this hypothesis. Here, participants were rather homogeneous in terms of attitudes and food habits, so these failed to explain the segmentation of participants in regards to their attitudes and habits associated with sweetener or cookie consumption. It has also been reported that it is not always possible to identify relationships between variables such as age, gender and education level with segmentation according to liking tendency because complex interactions between the “functional sweetener” context and other variables are involved in segmentation [55,56]. In addition, factors other than cookie- and sweetener-related dietary habits investigated in this study, such as consumer perception of health or indulgent food, might have influenced segmentation. Finally, it is possible that the number of samples constituting each cluster was insufficient to produce a statistically significant difference. Although more detailed information on participants’ attitudes was not collected in this study, it was possible to deduce the characteristics of clusters based on their responses and shifts in liking.

Considering the dramatic decrease in liking for SCL in Cluster 1, it seems that participants who were more focused on “natural” or “synthetic” information than “low-calories” or “functionality” were classified together. Since some consumers who care about health also value the use of natural ingredients in addition to low-sugar [57], “synthetic sweetener” information on sucralose might have decreased particularly their liking for SCL. Since only nine members comprised Cluster 1, this result requires verification using a larger number of subjects in future studies. In Cluster 2, the sensory pleasure of cookies seemed to precede health or other considerations. However, for the consumers in Cluster 3, it can be inferred that health considerations were a major determinant of acceptance ahead of taste. Consumers with a high interest in health are known to be more willing to give up the pleasure of taste for health than those with a low interest in health [58]. When information on health-promoting effects was provided, undesirable flavors, such as bitterness and astringency, may have been processed as cues that functional ingredients were contained in the food. Consequently, unpleasurable sensory characteristics may have been accepted more readily and could have had a positive impact on liking [29]. In addition, food neophobia has been reported as a predictor of consumer response to functional foods [59]. Although the neophobic tendency of participants was not investigated in the present study, suspicion toward novel or functional foods provides neophobic consumers with a reason for rejection [60], whereas neophilic consumers positively perceive and accept new food-related situations [61]. Therefore, it is possible that the different reactions of participants in Clusters 2 and 3 to the “functional sweetener” context may be partially attributed to a propensity for food neophobia.

In future studies, it will be necessary to investigate the moderating effect of the aforementioned variables using a broader and larger population to understand how information on health-promoting effects and sensory characteristics can influence individuals’ liking. In addition, by introducing appropriate control variables, such as food-neophobic propensity, food craving or BMI(body mass index), a systematic analysis of the process by which individual characteristics control the influence of information could be performed.

## 4. Conclusions

Replacement of sugar with alternative sweeteners resulted in undesirable sensory characteristics; however, these undesirable changes in sensory characteristics were improved by partial rather than full replacement. Additionally, developing a “functional sweetener” context by providing information on the health-promoting effects of alternative sweeteners contributed to some extent to improving liking by giving consumers a positive impression. Furthermore, consumers were able to discriminate samples better with regard to their liking when information was provided. These results support the hypothesis that hedonic response is influenced by non-sensory cognitive stimuli such as provision of health information. In addition, individuals were found to respond to the same information in different ways. However, demographic factors and food habits related to cookie consumption and alternative sweeteners could not explain why individuals reacted differently. Their hedonic responses implied that personal factors, for example, their health orientation, might have played a role. Therefore, it will be necessary to further study the variables that moderate the influence of the ”functional sweetener” context, as well as the interaction between the information and sensory characteristics, on the acceptance of sweeteners.

## Figures and Tables

**Table 1:** Sample information.

Sample	Sweetener	% Sucrose Substituted	Concentration ^1^ (% of Sweetener in Whole Cookie)
SUC	Sucrose	-	15.33
SCL	Sucralose	100	0.0302
XBS	Xylobiose + Sucrose	100	Xylobiose: 1.0728; Sucrose: 14.250
PHY100	Phyllodulcin	100	0.0460
PHY50	Phyllodulcin + Sucrose	50	Phyllodulcin: 0.0460; Sucrose: 7.665
PHX50	Phyllodulcin + Xylitol	100	Phyllodulcin: 0.0460; Xylitol: 7.665
RBA100	Rebaudioside A	100	0.0434
RBA50	Rebaudioside A + Sucrose	50	Rebaudioside A: 0.0217; Sucrose: 7.665

^1^ Concentrations of sweetener in cookie samples were determined using a preliminary 2-AFC test that evaluated the relative sweetness of each sweetener to that of sucrose in the cookie system.

**Table 2:** Basic formulation for the preparation of sugar cookies.

Ingredients	Manufacturer	Weight (g)	Flour Weight Basis (%)
Cookie flour	Soft weak flour, Qone Co., Gyeonggi-do, Korea	260	100
Butter	Unsalted butter, Seoul Milk Co., Seoul, Korea	130	50
Sugar	White sugar, TS Corporation, Seoul, Korea	80	30.7
Salt	Pure salt, Hanju Co., Ulsan, Korea	2	0.8
Egg yolk	Fertile chicken eggs, Heungsaeng poultry farming, Gyeongsangbuk-do, Korea	50	19.2

**Table 3:** Definitions and reference materials for descriptive attributes of sweetener cookie samples.

Attributes		Definition	Reference Materials	Scale Value (0–15)
Appearance	Brown color	Intensity of brown color	NCSS2040-Y30R (NCS color index, Scandinavian Color Institute AB, Stockholm, Sweden)	10
	Spots	Overall amount of small and large spots that appeared on the surface		
Odor	Roasted bean	Smell associated with roasted bean flour		
Taste	Sweetness	Fundamental taste sensation of which sucrose is typical	3% sucrose solution (Foodream refined white sugar, TS Co., Seoul, Korea)	7.5
	Saltiness	Fundamental taste sensation of which salt is typical	0.2% salt solution (Pure salt, Hanju Co., Ulsan, Korea)	8.5
	Umami	Fundamental taste sensation of which monosodium glutamate is typical	0.03% monosodium solution (Monosodium glutamate, Daesang Co., Seoul, Korea)	N/A ^1^
Flavor	Roasted bean	Aromatics associated with roasted bean flour		
Aftertaste	Sweetness	Fundamental taste sensation of which sucrose is typical after swallowing	See the reference for sweetness under taste category	
	Umami	Fundamental taste sensation of which monosodium glutamate is typical after swallowing	See the reference for umami under taste category	
	Roasted bean	Aromatics associated with roasted bean flour after swallowing		
	Acridness	The feeling of a sting on the tip of tongue after swallowing		
	Bitterness	Fundamental taste sensation of which caffeine and quinine are typical after swallowing		
Texture	Hardness	Force required to bite through using molars on the first 1 or 2 bites	Butterwaffles cookie (Crown Co., Seoul, Korea)	8
	Fractura-bility	Force with which the sample breaks when chewing 3 or 4 times between molars	Rusk (Honey Rusk, Samlip Co., Siheung, Korea)	3.5
	Hardness of fragment	Hardness of cookie fragments perceived after chewing 5 or 6 times	Whole grain wheat cookie (Diget, Orion Co., Seoul, Korea)	5
	Heterogeneity of particle size	Heterogeneity of particles of cookie samples perceived in the mouth after chewing 10–20 times	1 part perilla seed powder (Tureban Co., Goyang, Korea) in 2 parts water by weight	6

^1^ N/A shows that the reference standard of a sensory attribute does not have a specific scale value because the reference sample was only used for assessors’ concept alignment.

**Table 4:** Mean intensities of descriptive sensory attributes elicited by eight sweetener cookies.

	SUC	SCL	XBS	PHY100	PHY50	PHX50	RBA100	RBA50
Brown color	2.95 ^a,1^ (1.39) ^2^	1.20 ^b^ (0.69)	12.24 ^c^ (0.97)	4.40 ^d^ (1.67)	3.54 ^e^ (1.54)	7.53 ^f^ (1.62)	4.34 ^d^ (1.46)	4.00 ^d,e^ (1.50)
Spot	1.23 ^a^ (1.21)	0.45 ^b^ (0.60)	2.84 ^c^ (2.07)	1.51 ^a^ (1.39)	3.49 ^d^ (1.95)	9.38 ^e^ (1.56)	1.11 ^a^ (1.20)	3.58 ^d^ (1.81)
Roasted bean aroma	0.00 ^a^ (0.00)	0.00 ^a^ (0.00)	9.10 ^b^ (1.00)	0.00 ^a^ (0.00)	0.00 ^a^ (0.00)	0.00 ^a^ (0.00)	0.00 ^a^ (0.00)	0.00 ^a^ (0.00)
Sweetness	9.06 ^e^ (1.77)	8.96 ^d,e^ (1.97)	5.66 ^c^ (1.41)	0.58 ^a^ (0.98)	8.06 ^d,e^ (1.75)	6.11 ^c^ (2.43)	3.14 ^b^ (2.02)	7.90 ^d^ (1.74)
Saltiness	5.00 ^a^ (3.39)	5.73 ^a,b^ (3.22)	7.18 ^b^ (2.63)	8.29 ^d^ (3.68)	7.00 ^b,c^ (2.83)	6.64 ^b,c^ (3.33)	8.25 ^c,d^ (2.71)	6.49 ^a,b^ (3.46)
Umami	0.00 ^a^ (0.00)	0.10 ^a^ (0.30)	0.20 ^a^ (0.60)	7.45 ^b^ (2.39)	0.23 ^a^ (0.62)	0.00 ^a^ (0.00)	2.20 ^a,c^ (1.73)	0.10 ^a^ (0.38)
Roasted bean flavor	0.75 ^a^ (0.35)	0.00 ^b^ (0.00)	9.23 ^c^ (0.10)	0.00 ^b^ (0.00)	0.00 ^b^ (0.00)	0.00 ^b^ (0.00)	0.00 ^b^ (0.00)	0.00 ^b^ (0.00)
Sweetness aftertaste	7.85 ^a^ (2.67)	7.71 ^a^ (2.65)	5.09 ^b^ (1.94)	0.43 ^c^ (0.98)	7.00 ^a^ (2.40)	5.18 ^b^ (2.52)	2.48 ^d^ (2.06)	7.15 ^a^ (2.30)
Roasted bean flavor aftertaste	0.25 ^a^ (0.16)	0.00 ^b^ (0.00)	8.45 ^c^ (1.81)	0.00 ^b^ (0.00)	0.00 ^b^ (0.00)	0.00 ^b^ (0.00)	0.00 ^b^ (0.00)	0.00 ^b^ (0.00)
Umami aftertaste	0.00 ^a^ (0.00)	0.08 ^a^ (0.27)	0.10 ^a^ (0.30)	6.38 ^b^ (2.76)	0.23 ^a^ (0.62)	0.00 ^a^ (0.00)	1.45 ^c^ (1.58)	0.08 ^a^ (0.35)
Bitterness aftertaste	0.00 ^a^ (0.00)	0.03 ^a^ (0.16)	0.10 ^a^ (0.30)	5.56 ^b^ (3.60)	0.28 ^a^ (0.64)	0.03 ^a^ (0.16)	1.11 ^c^ (1.65)	0.03 ^a^ (0.16)
Acridness aftertaste	0.00 ^a^ (0.00)	0.03 ^a^ (0.16)	0.00 ^a^ (0.00)	7.48 ^b^ (1.78)	0.18 ^a^ (0.55)	0.00 ^a^ (0.00)	1.65 ^c^ (1.97)	0.03 ^a^ (0.16)
Hardness	2.30 ^a^ (8.80)	7.06 ^b^ (1.57)	2.10 ^a^ (1.00)	7.84 ^c^ (1.57)	5.49 ^d^ (1.62)	2.44 ^a^ (1.38)	6.85 ^b^ (1.75)	5.18 ^d^ (1.68)
Fracturability	2.14 ^a^ (0.90)	6.14 ^b,c^ (1.25)	1.98 ^a^ (0.82)	6.78 ^c^ (1.17)	4.61 ^d^ (1.61)	2.14 ^a^ (1.10)	6.00 ^b^ (1.63)	4.5 ^d^ (1.24)
Hardness of fragment	2.28 ^a^ (0.85)	6.86 ^b^ (1.28)	2.13 ^a^ (0.89)	7.51 ^c^ (0.98)	5.15 ^d^ (1.52)	2.16 ^a^ (0.99)	6.91 ^b,c^ (1.22)	5.06 ^d^ (1.42)
Heterogeneity of particle size	0.13 ^a^ (0.46)	0.00 ^a^ (0.00)	5.29 ^b^ (1.36)	0.00 ^a^ (0.00)	0.03 ^a^ (0.16)	0.10 ^a^ (0.63)	0.00 ^a^ (0.00)	0.00 ^a^ (0.00)

^1^ Different lowercase letters indicate significant differences among samples (*p* < 0.05). ^2^ Standard deviation.

**Table 5:** Consumer demographic profiles and cookie-related eating habits (n = 96).

Classification		Percentage (%)
Gender	Male	38.5
	Female	61.5
Age	19–29	52.1
	30–39	27.1
	40–49	20.8
Occupation	Student	53.1
	Homemaker	16.7
	Office worker	17.7
	Part-time worker	5.2
	Other	7.3
Disease	Hypertension	4.2
	Diabetes	1.0
	Hyperlipidemia	1.0
	None	93.8
Previous experience in weight control	No	25.0
	Experienced	58.3
	Ongoing	16.7
Previous exposures to low-sugar foods	Yes	62.5
	No	37.5
Criteria for food purchase	Flavor	77.1
	Ingredient	10.4
	Price	9.4
	Functionality	1.0
	Others	2.1
Cookie intake frequency	Every day	2.1
	More than once a week	19.8
	More than once a month but less than once a week	55.2
	More than 2 or 3 times a year but less than once a month	16.7
	Rarely	6.3

**Table 6:** *F* values and *p* values associated with the effects of sample, information and a two-way interaction between two factors on consumer acceptability in relation to eight cookie samples.

	Sample		Information		Sample	Information
	*F* Value	*p* Value	*F* Value	*p* Value	*F* Value	*p* Value
Overall liking	30.007	**<0.001 ^1^**	3.075	0.080	0.289	0.959
Appearance liking	11.502	**<0.001**	0.471	0.493	0.715	0.660
Flavor liking	23.586	**<0.001**	1.583	0.208	0.592	0.763
Texture liking	11.633	**<0.001**	0.042	0.838	0.465	0.860
Purchase intent	26.065	**<0.001**	13.819	**<0.001**	0.471	0.856

^1^*p* values < 0.05 are highlighted in bold.

**Table 7:** Mean score of consumer liking and purchase intent for eight cookie samples under different information conditions.

Sample	Overall Liking ^1^			Appearance Liking			Flavor Liking			Texture Liking			Purchase Intent		
	Mean ^2^	Blind	Info	Mean	Blind	Info	Mean	Blind	Info	Mean	Blind	Info	Mean	Blind	Info
SUC	6.0 ^a^	**5.9 ^a,3^**	**6.1 ^a^**	5.8 ^a^	**5.7 ^a^**	**5.9 ^a^**	6.0 ^a^	**5.9 ^a^**	**6.1 ^a^**	5.8 ^a,b^	5.8 ^a^	5.8 ^a^	4.4 ^a^	4.3 ^a^	4.6 ^a^
SCL	5.4 ^a,b^	**5.5 ^a,b^**	**5.4 ^b,c^**	5.6 ^a^	5.5 ^a,b^	5.7 ^a^	5.6 ^a,b,c^	5.7 ^a^	5.5 ^a,b,c^	5.4 ^a,b,c^	**5.5 ^a,b^**	**5.3 ^a,b,c^**	4.0 ^b^^,^^c^	3.9 ^a,b^	4.1 ^a,b,c^
XBS	5.1 ^b,c^	5.0 ^b,c^	5.2 ^c,d^	4.7 ^b^	4.8 ^b^	4.7 ^b^	5.2 ^c,d^	5.1 ^a,b^	5.4 ^b,c^	5.4 ^a,b,c^	5.4 ^a,b^	5.3 ^a,b,c^	3.8 ^b,c,d^	3.6 ^b,c^	4.1 ^a,b^
PHY100	3.9 ^d^	3.8 ^d^	4.1 ^e^	5.4 ^a^	5.3 ^a,b^	5.5 ^a^	4.1 ^e^	**3.9 ^c^**	**4.3 ^d^**	4.6 ^d^	**4.5 ^c^**	**4.8 ^c^**	2.7 ^e^	**2.5 ^d^**	**3.0 ^d^**
PHY50	5.5 ^a,b^	5.4 ^a,b^	5.5 ^a,b,c^	5.4 ^a^	5.5 ^a,b^	5.4 ^a^	5.4 ^a,b,c,d^	5.4 ^a,b^	5.5 ^a,b,c^	5.6 ^a,b,c^	5.7 ^a,b^	5.6 ^a,b^	4.0 ^b,c^	3.9 ^a,b^	4.2 ^a,b^
PHX50	5.1 ^b,c^	**5.0 ^b,c^**	**5.2 ^c,d^**	4.7 ^b^	4.8 ^b^	4.6 ^b^	5.3 ^b,c,d^	5.3 ^a,b^	5.2 ^b,c^	5.3 ^b,c,d^	5.2 ^a,b,c^	5.3 ^a,b,c^	3.7 ^c,d^	3.7 ^a,b,c^	3.8 ^b,c^
RBA100	4.5 ^c^	**4.5 ^c,d^**	**4.6 ^d,e^**	5.4 ^a^	5.3 ^a,b^	5.6 ^a^	4.9 ^d^	4.8 ^b^	4.9 ^c,d^	5.0 ^c,d^	4.9 ^b,c^	5.1 ^b,c^	3.3 ^d^	3.2 ^c^	3.5 ^c,d^
RBA50	5.8 ^a^	**5.6 ^a,b^**	**5.9 ^a,b^**	5.6 ^a^	5.5 ^a,b^	5.6 ^a^	5.8 ^a,b^	5.8 ^a^	5.9 ^a,b^	5.9 ^a^	5.9 ^a^	5.9 ^a^	4.3 ^b^	4.2 ^a,b^	4.5 ^a^

^1^ Means within a row that do not share a superscript letter are significantly different (*p* < 0.05, Tukey’s HSD test). ^2^ Means represent the average of two information condition scores. ^3^ Bold values indicate a significant different between the blind and informed (Info) conditions (*p* < 0.05, paired *t*-test).

**Table 8:** Consumer segmentation according to acceptance shift with the provision of information.

	SUC ^1^	SCL	XBS	PHY100	PHY50	PHX50	RBA100	RBA50
Cluster 1 (*n* = 9)	+0.56	–4.00 ^2^	–0.56	–2.22	–1.44	+1.22	–1.00	+1.44
Cluster 2 (*n* = 41)	+1.05	+0.80	+0.68	–0.11	+1.22	+1.63	+0.12	+0.61
Cluster 3 (*n* = 46)	–0.48	–0.04	−0.13	+1.13	–0.63	–1.26	+0.11	–0.28

^1^ The (+) indicates an increase in overall liking whereas the (–) indicates a decrease in overall liking after information was presented. ^2^ Bold values indicate a significant response shift (i.e., overall liking in informed condition–overall liking in blind condition ≠ 0) (*p* < 0.05, paired *t*-test).
